# Prevalence of selected cardiometabolic risk factors among adults in urban and semi-urban hospitals in four sub-Saharan African countries

**DOI:** 10.5830/CVJA-2016-072

**Published:** 2017

**Authors:** Samuel Kingue, Solofonirina Rakotoarimanana, Nirina Rabearivony, Francois Lepira Bompera

**Affiliations:** Department of Cardiology, Faculty of Medicine of Yaounde, General Hospital of Yaounde, Yaounde, Cameroon; Department of Cardiology, Joseph Raseta Defelatalala University Hospital, Antananariv, Madagascar; Department of Cardiology, Joseph Raseta Defelatalala University Hospital, Antananariv, Madagascar; Division of Nephrology, Department of Internal Medicine, University Clinic, Democratic Republic of Congo

**Keywords:** cardiovascular risk factors, metabolic syndrome, sub-Saharan Africa

## Abstract

**Aim:**

Cardiovascular diseases (CVDs) are a global challenge but the burden in sub-Saharan African (SSA) countries is less well documented than elsewhere. We aimed to describe the key cardiometabolic risk factors in four SSA countries.

**Methods:**

A cross-sectional, multi-national, hospital-based study was carried out among adults (> 35 years) across four SSA countries from 12 December 2011 to 7 February 2013. Risk factors were defined using the World Health Organisation and International Diabetes Federation guidelines.

**Results:**

Of the 844 adults (57.4% female, mean age 52.6 years), 76.6% were urban residents. The predominant CVD risk factors were hypertension (74.1%), obesity (36.2%) and excessive alcohol consumption (25.6%). Diabetes (17.7 vs 10.0%), obesity (42.8 vs 16.8%) and hypercholesterolaemia (25.8 vs 18.0%) were more prevalent among the hypertensive subjects (all p < 0.007) than the normotensives. The metabolic syndrome (39.4%) was more common in women and hypertensive subjects.

**Conclusions:**

Hospital patients in SSA countries present with excessive rates of cardiometabolic risk factors. Focus on their prevention and control is warranted.

## Aim

Non-communicable diseases (NCDs) are rapidly increasing in incidence in sub-Saharan Africa (SSA). Cardiovascular disease (CVD) is the leading contributor to the global burden of NCDs.^1^ Hypertension, which is the main driver of CVD, has been estimated to affect about 972 million adults worldwide, a figure that is projected to increase by 60% by the year 2025.^2^,^3^ This high prevalence of hypertension is coupled with poor detection, treatment and control rates.^4^

Diabetes mellitus is also a leading cause of morbidity and mortality from NCDs and a major precursor of CVD.^5^ The population of people with diabetes in SSA is growing more rapidly than anywhere else, and is expected to nearly double within the next two decades.^6^ The co-occurrence of diabetes and hypertension in the same individual compounds the harmful effects of each condition.

A recent cross-sectional study conducted in semi-urban Cameroon has indicated the co-occurrence of diabetes and hypertension, affecting up to 5% of adults.^7^ Other common drivers of NCDs and the CVD burden include physical inactivity, smoking, unhealthy diet, dyslipidaemia, excess weight and alcohol abuse.^8^,^9^

Monitoring the risk profile of the population is an extremely important component of the strategy to prevent and control NCDs in general and CVD in particular. This pivotal role was recently highlighted in the World Health Organisation (WHO) global action plan of 2013–2020 for the prevention of NCDs.^10^ Given the silent nature of hypertension and other risk factors, and the lack of awareness of them in low- and middle-income countries (LMICs), opportunistic screening and awareness have been highlighted by the World Heart Federation as the key first steps to improving management and prevention.^11^

Studies addressing the risk profile of individuals who have contact with hospitals in Africa are lacking, and most of the existing studies are single-country studies, therefore offering less opportunity to examine between-country variabilities. This report is on a multi-country, multi-centre, health facilities-based study to assess the distribution of major cardiometabolic risk factors in adults in urban settings across different countries in SSA.

## Methods

This was a multi-national, multi-centre, cross-sectional study conducted from 12 December 2011 to 7 February 2013. The following SSA countries participated in the study: Cameroon (13 centres), Nigeria (five centres), Democratic Republic of Congo (DRC) (11 centres) and Madagascar (24 centres). The study centres were purposefully selected from the health districts of the capital cities (urban and semi-urban) in the participating countries. Participating centres included both public and private healthcare facilities. General practitioners working in the selected centres were trained to consecutively recruit all individuals aged over 35 years to their facilities, regardless of the reason for the visit to hospital, if they were resident in the particular city for at least three months.

Ethical approval was obtained from the ethics committees of the participating countries and the patients gave written consent before enrollment. The study complied with the Declaration of Helsinki.

Data were collected simultaneously in all study centres of the participating countries, using a standardised case report form (CRF). The following variables were collected: sociodemographic characteristics (age, gender, educational level, alcohol consumption, tobacco use and employment type), history of hypertension, diabetes status and systolic and diastolic blood pressure (BP, in mmHg).

BP was measured using an automated BP machine (Omron 750 IT) in the seated position after the participant had been at rest for five to 10 minutes. Three measurements were taken on the right arm and the average of the last two was retained.^12^ Weight, height, and waist and hip circumference were measured using standard procedures and equipment following WHO guidelines.^13^ Weight was measured to the nearest 0.5 kg and height to the nearest 0.5 cm. Body mass index (BMI, kg/m^2^) was calculated as body weight in kg divided by the square of the height in metres. The waist circumference (WC) was measured with a tape midway between the lower rib margin and iliac crest. Waist-to-hip ratio was calculated as waist circumference (cm) divided by hip circumference (cm).

Fasting capillary glucose concentration was obtained using a standardised glucometer (Accu-chek Aviva; Hoffmann-LA Roche, Ltd, Germany) in all the settings. Fasting total cholesterol, high-density lipoprotein (HDL-C) and low-density lipoprotein cholesterol (LDL-C), triglycerides, uric acid and serum creatinine concentrations were acquired using locally available routine standard techniques and procedures.

Hypertension was diagnosed in the presence of systolic or diastolic blood pressure ≥ 140 or 90 mmHg or ongoing blood pressure-lowering medications over the past 15 consecutive days. Uncontrolled hypertension was defined as blood pressure ≥ 140/90 mmHg in participants on BP control agents for the last 30 consecutive days. Duration of hypertension was defined as date of survey minus date of diagnosis of hypertension.

Hyperglycaemia was defined as fasting capillary glucose level ≥ 6.1 mmol/l (110 mg/l) and diabetes was defined as fasting capillary glucose level ≥ 7.1 mmol/l (126 mg/dl) orphysician-documented history of diabetes, or patient on glucosecontrolling agents (oral or insulin) for the last 15 consecutive days. Impaired fasting glycaemia was defined as fasting capillary glucose levels between 6.1 and 7.1 mmol/l (110–126 mg/dl).

Overweight and obesity were defined using BMI and WHO criteria,^14^ i.e. normal: 18.5 kg/m^2^ ≤ BMI ≤ 24.99 kg/m^2^; overweight: 25 kg/m^2^ ≤ BMI ≤ 29.99 kg/m^2^; obesity: 30 kg/m^2^ ≤ BMI ≤ 39.99 kg/m^2^, morbid obesity: BMI ≥ 40 kg/m2. Hypercholesterolaemia was defined as a total cholesterol level > 5.18 mmol/l.

The metabolic syndrome (MS) was defined according to the International Diabetes Federation (IDF) consensus criteria:15 central obesity plus any two of the following: raised triglyceride levels ≥ 150 mg/dl (1.7 mmol/l) or specific treatment for this lipid abnormality, reduced HDL-C < 40 mg/dl (1.03 mmol/l) in men and < 50 mg/dl (1.29 mmol/l) in women or specific treatment for this lipid abnormality, raised blood pressure (≥ 130/85 mmHg) or treatment of previously diagnosed hypertension, raised fasting plasma glucose level ≥ 100 mg/dl (5.6 mmol/l) or previously diagnosed type 2 diabetes.^15^

Increased waist circumference was defined as > 102 cm for men and > 88 cm for women. With a BMI > 30 kg/m^2^, central obesity was assumed without measurement of waist circumference.^15^

Alcohol consumption was categorised as low-to-moderate consumption (less than or equal to one local beer daily for women and two local beers for men) and excessive consumption (more than two local beers daily).^16^ Smoking status was determined as current smokers, former smokers (having smoked in the past but having stopped for two or more weeks prior to the survey, however, those who had stopped within two weeks of the survey were considered current smokers), and never smoked.

## Statistical analysis

Data analysis was done using the Statistical Package for Social Sciences (SSPS Inc, Chicago, IL) software version 20.0. Categorical variables were summarised as counts and percentages while continuous variables were summarised as means, median, standard deviation (SD) and percentiles where appropriate. Group comparisons used the chi-squared or Fisher’s exact tests for categorical variables, and the Student t-test for continuous variables. A p-value < 0.05 was considered statistically significant.

## Results

Table 1 shows the general characteristics of the study population. A total of 844 adults (57.4% were women and overall mean age was 52.6 ± 11.6 years) were included in the study, among whom 154 and 216, respectively, were from Cameroon and Nigeria, 240 from the DRC and 240 from Madagascar. The majority (76.6%) of the study participants were urban dwellers. The men were more likely to be employed and to be educated than the women (both p < 0.001). The women were more likely to be overweight, obese or morbidly obese than the men (p < 0.001). The men had a significantly higher mean triglyceride levels than the women (2.9 vs 2.2 mmol/l; p = 0.019) and lower mean HDL-C levels (1.6 vs 1.8 mmol/l; p = 0.004). Men also had higher mean normal values of serum creatinine (90.8 vs 75.7 μmol/l, p < 0.001) and uric acid (295.0 vs 233.2 μmol/l, p < 0.001) than the women. Men and women had similar mean systolic (149.5 vs 149.5 mmHg) and diastolic (91.9 vs 90.6 mmHg) blood pressures, respectively.

**Table 1:** General characteristics and overall profile of study population

**	*Cameroon (n = 154) 17.9%*			*Nigeria (n = 211) 25.9%*			*DRC (n = 239) 28.1%*			*Madagascar (n = 240) 28.1%*			*Total (n = 844)*			**
Variables	Male (n = 54)	Female (n = 100)	p-value	Male (n = 87)	Female (n = 124)	p-value	Male (n = 116)	Female (n = 123)	p-value	Male (n = 103)	Female (n = 137)	p-value	Male (n = 360) (42.6%)	Female (n = 484) (57.4%)	Total (n = 844)	p-value
Age (years)	51.5 ± 9.7	56.4 ± 11.2	0.009	50.3 ± 13.2	48.8 ± 11.3	0.369	56.7 ± 11.2	55.7 ± 12.9	0.532	51.4 ± 10.0	49.5 ± 9.9	0.147	52.8 ± 11.5	52.3 ± 11.8	52.6 ± 11.7	0.512
BMI (kg/m2)	29.7 ± 3.8	31.1 ± 6.7	0.184	28.6 ± 6.1	31.2 ± 6.4	0.003	27.7 ± 6.8	30.4 ± 6.2	0.002	25.1 ± 4.0	25.0 ± 3.9	0.930	27.5 ± 5.8	29.3 ± 6.4	28.5 ± 6.1	0.000
BMI category, n (%)																
Normal	06 (11.1)	22 (22.0)		29 (33.4)	21 (16.8)		39 (33.6)	25 (20.4)		53 (51.4)	69 (50.4)		127 (35.3)	137 (28.3)	264 (31.3)	
Overweight	22 (40.7)	23 (23.0)	0.005	27 (31.0)	32 (25.8)	0.007	38 (32.8)	37 (30.1)	0.005	40 (38.8)	54 (39.4)	0.983	127 (35.3)	146 (30.2)	273 (32.3)	0.000
Obese	26 (48.2)	43 (43.0)		28 (32.2)	59 (47.7)		38 (32.8)	50 (40.6)		10 (9.7)	14 (10.2)		102 (28.3)	166 (34.3)	268 (31.8)	
Morbidly obese	00 (0.0)	12 (12.0)		03 (3.4)	12 (9.7)		01 (0.8)	11 (8.9)		00 (0.0)	00 (0.0)		04(1.1)	35 (7.2)	39 (4.6)	
WC (cm)	101.2 ± 12.8	102.7 ± 16.1	0.559	91.2 ± 11.5	97.4 ± 15.4	0.001	97.7 ± 17.9	99.9 ± 14.5	0.279	91.2 ± 11.2	88.4 ± 10.5	0.049	94.8 ± 14.5	96.6 ± 15.1	95.8 ± 14.8	0.071
HC (cm)	104.5 ± 12.3	109.4 ± 19.8	0.101	96.8 ± 12.6	106.2 ± 18.2	0.000	101.1 ± 10.0	110.6 ± 14.6	0.000	97.8 ± 7.5	99.4 ± 9.1	0.155	99.6 ± 10.8	106.1 ± 16.2	103.2 ± 14.5	0.000
SBP (mmHg)	168.5 ± 17.4 167.9 ± 17.8	167.9 ± 17.8	0.855	138.8 ± 24.8	140.4 ± 25.7	0.656	163.6 ± 15.8	166.4 ± 21.2	0.260	132.3 ± 22.7	128.7 ± 23.1	0.231	149.5 ± 25.5	149.5 ± 27.9	149.3 ± 26.9	0.981
DBP (mmHg)	102.9 ± 11.4	100.4 ± 12.2	0.221	84.8 ± 11.8	86.2 ± 14.4	0.462	98.2 ± 11.5	96.4 ± 11.3	0.248	84.8 ± 13.9	82.2 ± 15.2	0.183	91.9 ± 14.4	90.6 ± 15.3	91.1 ± 14.9	0.239
History of HTN, n (%)																
Yes	01 (1.9)	05 (5.1)	0.423	50 (57.5)	82 (66.1)	0.248	81 (69.8)	93 (75.6)	0.383	38 (36.9)	55 (40.1)	0.688	170 (47.2)	235 (48.8)	405 (48.1)	0.729
No	53 (98.1)	93 (94.9)		37 (42.5)	42 (33.9)		35 (30.2)	30 (24.4)		65 (63.1)	82 (59.9)		190 (52.8)	247 (51.2)	437 (51.9)	
>History of DM, n (%)																
Yes	06 (11.1)	09 (9.1)	0.778	10 (11.9)	12 (9.7)	0.820	11 (9.6)	14 (11.5)	0.677	06 (5.9)	02 (1.5)	0.077	33(9.3)	37(7.8)	70 (8.4)	0.452
No	48 (88.9)	90 (90.9)		74 (88.1)	104 (90.3)		104 (90.4)	108 (88.5)		97 (94.1)	135 (98.5)		323(90.7)	437(92.2)	760 (91.6)	
FBS (mmol/l)	6.2 ± 2.8	6.6 ±7.6	0.685	6.2 ±2.9	5.8 ± 4.1	0.979	5.4 ± 1.8	5.1 ± 1.6	0.084	5.9 ± 2.0	5.5 ± 1.6	0.122	5.8 ± 2.3	5.7 ± 4.2	5.8 ± 3.5	0.798
Lipid profile																
TG (mmol/l)	2.4 ± 10.0	0.9 ± 0.5	0.144	6.8 ± 5.8	5.8 ± 4.1	0.173	1.1 ± 0.6	1.0 ± 0.4	0.266	2.1 ± 1.4	1.4 ± 0.8	0.000	2.9 ± 5.3	2.2 ± 2.8	2.5 ± 4.1	0.019
HDL-C (mmol/l)	1.2 ± 0.5	1.3 ± 0.7	0.560	2.6 ± 1.1	2.9 ± 1.2	0.075	1.2 ± 0.3	1.4 ± 0.5	0.000	1.3 ± 0.6	1.5 ± 0.3	0.052	1.6 ± 0.9	1.8 ± 0.9	1.7 ± 0.9	0.004
LDL-C (mmol/l)	2.8 ± 0.8	2.5 ± 0.8	0.027	6.1 ± 3.3	6.5 ± 3.4	0.412	2.8 ± 1.2	2.9 ± 1.2	0.349	2.5 ± 0.8	2.7 ± 0.9	0.085	3.4 ± 2.3	3.6 ± 2.4	3.5 ± 2.4	0.436
TC (mmol/l)	4.4 ± 0.9	4.2 ± 0.9	0.317	8.8 ± 4.5	9.2 ± 4.8	0.549	4.5 ± 0.9	4.7 ± 1.1	0.154	4.8 ± 0.9	4.9 ± 1.1	0.360	5.5 ± 2.8	5.7 ± 3.0	5.6 ± 3.0	0.471
Creatinine (μmol/l)	108.6 ± 35.4	99.2 ± 40.4	0.153	56.2 ± 21.2	54.5 ± 28.4	0.658	107.2 ± 43.1	84.9 ± 22.1	0.000	88.8 ± 17.2	66.4 ± 11.4	0.000	90.8 ± 37.3	75.7 ± 31.1	82.1 ± 34.6	0.000
Uric acid (μmol/l)	384.6 ± 84.2	298.6 ± 119.9	0.000	300.7 ± 275.4	264.5 ± 242.1	0.350	405.3 ± 109.5	354.7 ± 100.1	0.000	340.6 ± 90.3	249.9 ± 67.9	0.000	295.0 ± 178.5	233.2 ± 154.1	259.3 ± 168.0	0.000
Alcohol intake, n (%)																
Low–moderate	22 (46.8)	49 (75.3)	0.003	–	–	–	52 (70.3)	45 (91.8)	0.006	38 (76.0)	20 (100.0)	0.014	114 (65.9)	115 (85.1)	229 (74.4)	0.000
Excessive	25 (53.2)	16 (24.7)					22 (29.7)	04 (8.2)		12 (34.0)	00 (0.0)		59 (34.1)	20 (14.9)	79 (25.6)	
Tobacco use, n (%)																
Current	05 (9.3)	05 (5.1)	0.004	04 (4.6)	00 (0.0)	0.000	10 (8.7)	01 (0.8)	0.000	26 (25.2)	22 (16.1)	0.007	45 (12.5)	28 (5.7)	73 (8.7)	0.000
Former	08 (14.8)	02 (2.0)		17 (19.7)	00 (0.0)		14 (12.2)	01 (0.8)		19 (18.4)	12 (8.7)		58 (16.1)	15 (3.1)	73 (8.7)	
Never	41 (75.9)	92 (92.9)		65 (75.7)	122 (100.0)		91 (79.1)	121 (98.4)		58 (56.3)	103 (75.2)		255 (71.4)	438 (91.2)	693 (82.6)	
Employment, n (%)																
Unemployed	0 (0.0)	14 (14.3)		01 (1.1)	06 (4.5)		12 (10.4)	16 (13.0)		02 (1.9)	06 (4.4)		15(4.1)	42(8.6)	57 (6.8)	
Full time	24 (44.4)	22 (22.4)		22 (24.4)	44 (35.3)		60 (51.7)	18 (14.6)		52 (50.5)	71 (51.8)		158(43.5)	155(31.8)	313 (37.2)	0.000
Part time	05 (9.3)	02 (2.0)	0.000	03 (3.3)	01 (0.8)	0.010	01 (0.8)	00 (0.0)	0.000	07 (6.8)	06 (4.4)	0.001	16(4.4)	09(1.9)	25 (2.9)	
Self-employed	13 (24.1)	09 (9.3)		46 (53.4)	50 (40.1)		24 (20.7)	18 (14.6)		29 (28.2)	24 (17.5)		112 (31.4)	101(21.4)	213 (25.3)	
Housewife	00 (0.0)	44 (44.9)		00 (0.0)	09 (7.2)		00 (0.0)	63 (51.2)		00 (0.0)	19 (13.8)		01 (0.3)	135(27.8)	136 (16.2)	
Retired	12 (22.2)	07 (7.1)		15 (17.8)	15 (12.1)		19 (16.4)	08 (6.5)		13 (12.6)	11 (8.0)		58 (16.3)	40(8.5)	98 (11.6)	
Education, n (%)																
None	01 (1.9)	15 (15.3)		01 (2.2)	11 (8.9)		02 (1.7)	18 (14.6)		00 (0.0)	01 (0.7)		04 (1.2)	45 (9.4)	49 (5.8)	
Primary school	12 (22.2)	38 (38.7)	0.000	54 (61.1)	70 (56.9)	0.113	05 (4.3)	31 (25.2)	0.000	11 (10.7)	12 (8.8)	0.024	82 (22.8)	151 (31.4)	233 (27.7)	0.000
High school	18 (33.3)	25 (25.5)		09 (10.0)	14 (11.4)		40 (34.5)	42 (34.1)		19 (18.4)	51 (37.2)		86 (23.8)	132 (27.4)	218 (25.9)	
Diploma	08 (14.8)	15 (15.3)		00 (0.0)	00 (0.0)	26 (22.4)	18 (14.6)	25 (24.3)	27 (19.7)		59 (16.4)	60 (12.5)	119 (14.1)	
Degree	15 (27.8)	05 (5.2)		23 (26.7)	28 (22.8)		43 (37.1)	14 (11.4)		48 (46.6)	46 (33.6)		129 (35.8)	93 (19.3)	222 (26.5)	
Residence, n (%)																
Urban	47 (87.0)	81 (82.6)	0.643	45 (51.7)	70 (56.9)	0.484	113 (97.4)	120 (97.5)	0.942	71 (68.9)	100 (72.9)	0.565	276 (76.6)	371 (77.1)	647 (76.9)	0.988
Semi-urban	07 (13.0)	17 (17.4)		42 (48.3)	53 (43.1)		03 (2.6)	03 (2.5)		32 (31.1)	37 (27.1)		84 (23.4)	110 (22.9)	194 (23.1)	

BMI = body mass index; WC = waist circumference; HC = hip circumference; SBP = systolic blood pressure; DBP = diastolic blood pressure; DM = diabetes mellitus; HTN = hypertension; FBS = fasting blood sugar; TG = triglycerides; HDL-C = high-density lipoprotein cholesterol; LDL-C = low-density lipoprotein cholesterol; TC = total cholesterol; DRC = Democratic Republic of Congo; p-values are for comparison between gender among participating countries.

The overall prevalence of hypertension [previously aware/diagnosed (48.1%) and newly diagnosed (26%)] was 74.1% [Cameroon (91.5%), Nigeria (66.8%), DRC (99.1%) and Madagascar (45.0%)]. The overall prevalence of diabetes in the study was 15.7% and ranged from 24.8% in Nigeria, 15.6% in Cameroon and 15.0% in DRC, to 8.7% in Madagascar (p = 0.003). Excessive alcohol consumption was reported in 25.6% of study participants, with the highest prevalence in Cameroon (36.6%), and the lowest in Nigeria, where all participants reported low-to-moderate consumption (p = 0.007).

Of the study participants, 17.3% were either current or former smokers. A significant difference (p < 0.001) in prevalence of smoking across the countries was noted, with the highest prevalence in Madagascar (32.9%), followed by Cameroon (13%), then DRC (10.9%), and Nigeria (10.0%) being the lowest.

Of the study participants, 32.3 and 36.3% were overweight and obese (obesity 31.8%), or morbidly obese (4.5%), respectively. Overweight was highest in Madagascar (39.2%) and lowest in Nigeria (28.4%), while overall obesity was highest in Cameroon (53.6%) and lowest in Madagascar (10.0%) (p < 0.001). Details of the cardiometabolic risk factors across the countries are shown in Table 2.

**Table 2:** Prevalence of selected risk factors across participating countries

*Risk factor*	*Cameroon n (%)*	*Nigeria n (%)*	*DRC n (%)*	*Madagascar n (%)*	*Total n (%)*	*p-value*
Hypertension (n = 844)						
Yes	141 (91.5)	141 (66.8)	237 (99.1)	108 (45.0)	630 (74.1)	0.000
No	13 (8.5)	70 (33.2)	02 (0.9)	132 (55.0)	220 (25.9)	
Diabetes (n = 839)						
Yes	24 (15.6)	51 (24.8)	36 (15.0)	21 (8.7)	132 (15.7)	0.000
No	130 (84.4)	154 (75.2)	204 (85.0)	219 (91.3)	707 (84.3)	
Alcohol consumption (n = 309)						
Low–moderate	71 (63.4)	03 (100.0)	98 (79.0)	58 (82.8)	230 (74.4)	0.007
Excessive	41 (36.6)	00 (0.0)	26 (21.0)	12 (17.2)	79 (25.6)	
Smoking (n = 844)						
Current	10 (6.5)	04 (1.8)	11 (4.6)	48 (20.0)	73 (8.6)	0.000
Former	10 (6.5)	18 (8.5)	15 (6.3)	31 (12.9)	74 (8.7)	
Never	133 (87.0)	190 (89.7)	213 (89.1)	161 (67.1)	697 (82.7)	
Obesity (n = 844)						
Normal	28 (18.2)	53 (25.1)	64 (26.7)	122 (50.8)	267 (31.6)	
Overweight	45 (29.2)	60 (28.4)	76 (31.6)	94 (39.2)	275 (32.6)	0.000
Obese	69 (44.8)	85 (40.3)	88 (36.7)	24 (10.0)	266 (31.5)	
Morbidly obese	12 (8.8)	13 (6.2)	11 (5.0)	00 (0.0)	36 (4.3)	

p-values = comparison of variables across countries.

When participants were assessed according to their hypertension status (Table 3), diabetes mellitus and hypercholesterolaemia were more common among participants with hypertension (17.7 vs 10.0%; p = 0.010 and 25.8 vs 18.0%; p = 0.008, respectively) than in normotensives. With regard to WC, 73.4% of women had a WC > 88 cm, most (79.4%) of whom were hypertensive, opposed to 67.7% who were non-hypertensive (p = 0.004). This difference was not statistically significant among the men. Overall, participants with hypertension were more likely to be overweight (33.2 vs 30.5%) and obese (42.5 vs 16.8%) (p < 0.001), but reported lower smoking rates (15.0 vs 24.1%; p = 0.002).

**Table 3:** Risk factors according to hypertension status in the study participants

*Variable*	*Hypertensives n (%)*	*Nonhypertensives n (%)*	*Total (n = 844) n (%)*	*p-value*
Tobacco smoking (n = 844)				
Current	42 (6.7)	31 (14.1)	73 (8.6)	0.002
Former	52 (8.3)	22 (10.0)	74 (8.8)	
Never	530 (85.0)	167 (75.9)	697 (82.6)	
Alcohol consumption (n = 309)				
Low to moderate	195 (73.6)	35 (79.5)	230 (74.4)	0.460
Excessive	70 (26.4)	09 (20.5)	79 (25.6)	
Obesity (n = 844)				
Normal	152 (24.3)	115 (52.7)	267 (31.5)	
Overweight	208 (33.2)	67 (30.5)	275 (32.5)	0.000
Obese	232 (37.1)	34 (15.9)	266 (31.5)	
Morbidly obese	34 (5.4)	02 (0.9)	36 (4.5)	
Waist circumference (n = 486)				
Men (> 102 cm)	76 (31.9)	86 (34.7)	162 (33.3)	0.564
Women (> 88 cm)	189 (79.4)	168 (67.7)	357 (73.4)	0.004
Diabetes mellitus (n = 839)				
Yes	110 (17.7)	22 (10.0)	132 (15.7)	0.007
No	509 (82.3)c	198 (90.0)	707 (84.3)	
Hypercholesterolaemia (n = 811)				
Yes	102 (25.8)	75 (18.0)	177 (21.8)	0.008
No	293 (74.2)	341 (82.0)	634 (78.2)	

p-value = comparison of variables between the two groups.

Hypertension and smoking were more prevalent in participants with diabetes (83.3 vs 71.9%; p = 0.010 and 19.7 vs 17.9%; p = 0.019, respectively). The distribution of other risk factors, including hypercholesterolaemia, increased WC, overweight and obesity, and excessive alcohol consumption, was not significantly different between the participants with and without diabetes. Details of the risk factors according to presence or absence of diabetes are shown in Table 4.

**Table 4:** Risk factors according to diabetes status in the study participants

*Variable*	*Diabetics n (%)*	*Nondiabetics n (%)*	*Total n (%)*	*p-value*
Tobacco smoking (n = 834)				
Current	07 (5.3)	66 (9.4)	73 (10.6)	
Former	19 (14.4)	54 (7.9)	73 (10.6)	0.019
Never	106 (80.3)	582 (82.7)	582 (82.7)	
Alcohol consumption (n = 308)				
Alcohol consumption	(n = 308)	199 (75.0)	229 (75.1)	0.456
Excessive	13 (30.2)	66 (25.0)	79 (24.9)	
Obesity (n = 839)
Normal	37 (28.0)	228 (32.2)	265 (31.6)	
Overweight	42 (31.8)	228 (32.2)	270 (32.2)	0.462
Obese	44 (33.3)	222 (31.6)	266 (31.7)	
Morbidly obese	09 (6.9)	29 (4.0)	38 (4.5)	
Waist circumference				
Men (> 102 cm) (n = 359)	10 (29.4)	85 (26.1)	95 (26.4)	0.685
Women (> 88 cm) (n = 478)	29 (76.3)	319 (72.5)	348 (72.8)	0.706
Hypertension (n = 839)
Hypertension (n = 839)	110 (83.3)	509 (71.9)	619 (73.7)	0.007
No	22 (16.7)	198 (28.1)	220 (26.3)	
Hypercholesterolaemia (n = 809)				
Yes	15 (22.1)	161 (21.7)	176 (21.8)	0.949
No	53 (77.9)	580 (78.3)	633 (78.	

Diabetics = participants with diabetes mellitus; non-diabetics = participants without diabetes mellitus; p-values = comparison of variables between both groups.

**Fig. 1.**
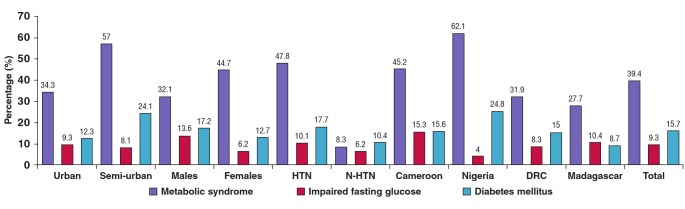
Prevalence of the metabolic syndrome, impaired fasting glucose levels and diabetes across countries, urbanicity, gender and hypertension status. HTN = hypertensives, N-HTN = non-hypertensives

The overall prevalence of the MS, impaired fasting glucose levels (IFG) and diabetes mellitus was 39.4, 9.3 and 15.7%, respectively. Detailed prevalences of the MS, IFG and diabetes according to urban or rural and hypertensive status, gender and country are presented in Fig. 1. The highest prevalence of the MS was reported in Nigeria (62.1%), then Cameroon (45.2%), DRC (31.9%), and the lowest in Madagascar (27.7%).

IFG was most prevalent in Cameroon (15.3%), followed by Madagascar (10.4%), DRC (8.3%) and Nigeria (4.0%). Nigeria had the highest prevalence of diabetes (25.0%), then Cameroon (15.6%), DRC (15.0%) and finally Madagascar (8.7%). Both the MS and IFG were more prevalent in hypertensive patients than in non-hypertensive subjects (47.8 vs 8.3% and 10.1 vs 6.2%, respectively).

Comparing gender, the MS was more prevalent in females (44.7 vs 32.1%), but incidence of IFG and diabetes was higher in males (13.6 and 17.2%) compared to females (6.2 and 12.7%), respectively. With regard to urban and rural status, the MS and diabetes were more prevalent in semi-urban dwellers (57 and 24.1%), opposed to urban dwellers (34.3 and 12.3%, respectively).

## Discussion

In this self-selected group of participant in a hospital-based study of cardiometabolic risk factors among adults in four SSA countries, we found a high prevalence of the MS, IFG and diabetes mellitus in all countries. In spite of the differences observed between countries, which may reflect differences in healthcare access and resources, and possibly selection bias, these findings clearly signify the rapid growth of cardiovascular risk factors in a region of the world that has traditionally been known as the hotspot of nutritional and infectious diseases. This study is therefore relevant for understanding the epidemiology of cardiovascular and metabolic risk profiles of adults in the region, a pivotal step in the control of the incidence of CVDs.

The overall prevalence of the MS in our study population was 39.4% and ranged from 62.1% in Nigeria to 27.7% in Madagascar. This was particularly for hypertensive subjects, female participants and semi-urban dwellers. The overall prevalence of the MS was lower than reported in Ghana among hypertensive patients. It was however similarly observed that the MS was more prevalent among women than men (OR: 4.88, p = 0.027) in this study.^17^ Another study among newly diagnosed type 2 diabetes subjects revealed higher prevalences of the MS of 68 and 81%, using IDF and WHO criteria, respectively. Again, as in our study, the MS was common in women and was driven essentially by female gender, family history of diabetes, overweight and obesity.^18^

IFG overall prevalence was 9.3% and ranged from 15.3% in Cameroon to 4.0% in Nigeria. Our findings are however higher than reported in a community-based study in South Africa,^19^ and Nigeria.^20^ These differences could be accounted for by the differences in study types (hospital based vs community based) and also geographical variations in the populations studied. However, the high prevalence of IFG among the participants is significant, as this represents a group of individuals at increased risk for transition to higher cardiovascular risk and the eventual development of diabetes if not properly controlled with lifestyle and dietary modifications.

Recent publications have highlighted the rapidly increasing prevalence of hypertension, coupled with under-diagnosis, undertreatment and low control rates in SSA.^4^,^20^,^21^ The high prevalence of hypertension in our hospital-based study and the fact that 25.8% of these patients were newly diagnosed or undiagnosed cases is therefore not surprising. The situation was similar with diabetes mellitus, with an overall prevalence of 15.7%, with 6.9% being undiagnosed cases, as previously described.^22^

In a recent meta-analysis that focused on the burden of hypertension in Africa,4 the pooled prevalence was 30%. Our prevalence is equivalent to the highest prevalence of 70% in the pooled studies. Another recent population-based study in Cameroon^21^ reported a prevalence of 47.5%, which was lower than reported in our cohort.

The CLARIFY registry, which explored geographical variations in cardiovascular risk factors among coronary artery disease (CAD) patients, reported a high prevalence of hypertension of 48% in Eastern Europe.^23^ The differences observed in these studies and others could be due to differences in populations studied and methodologies employed. Previous regional-based studies using similar methodology to ours are non-existent, therefore limiting the possibility for adequate comparison.

The high prevalence of diabetes in our study (15.7%) was slightly below the 17% noted among CAD patients in Eastern Europe but far lower than the 60% in the Middle East.^23^ While we acknowledge the dearth of African regional data on diabetes, some national studies are worth noting. The highest prevalence of diabetes among participating countries was from Nigeria, with a prevalence rate of 24.8%. This was lower than the 28.2% noted in a community-based study in South Africa,^19^ but higher than the 10.1% reported in a self-selected population study in Cameroon.^22^ Variations in degree of urbanisation, and differences in lifestyle, environmental factors and study settings (population vs hospital based) as well as sample sizes most likely account for the differences seen in these studies.

Overall mean BMI of our study participants was 28.5 kg/m^2^, which was higher than reported in Benin,^8^ although it was lower than reported in Ghana among hypertensive subjects.^17^ About one in three of the study participants was overweight or obese. This is likely to be explained by the increasing adoption of Western lifestyles, especially in urban areas (which were in the majority in our study), limited physical activity and increased sedentary lifestyles, which are wrongly attributed to good living. Similarly, a high prevalence of obesity has been reported in other parts of Africa,^8^,^17^,^24^ in relation to urbanisation and high socio-economic status.^25^ A community-based study in Cameroon by Fezeu and colleagues in 2010 demonstrated the influence of ethnicity and urbanisation on abdominal adiposity and obesityrelated abnormalities.^26^

A quarter of participants reported excessive alcohol consumption, and approximately one in five was either a current or former smoker. This is similar to the 19% smoking prevalence reported in Eastern Europe.^23^ These are well-established drivers for CVD,^27^ metabolic and other NCDs and most likely account in part for the high rates of hypertension, diabetes and obesity in our cohort. Our findings are supported by a recent meta-analysis of prospective studies on the association of alcohol consumption and CVD risk and mortality, where it was found that low-to-moderate alcohol consumption was inversely significantly associated with the risk of CVD and all-cause mortality among hypertensive patients.^27^

Risk profiles of the participants were examined according to hypertension status. A high prevalence of diabetes (17.7%) was noted among the hypertensive subjects, compared to 10% in non-hypertensives. This was half that reported in Ghana among hypertensive subjects,^17^ although higher than the 13.5% reported in Cameroon.^28^ Hypertensive patients were also more likely to be overweight and obese than non-hypertensive subjects, with prevalence rates of 33.1 and 42.8%, compared to 30.5 and 16.8%, respectively.

All other studied risk factors, such as hypercholesterolaemia, abdominal adiposity (WC > 88 cm for women and 102 cm for men), and excessive alcohol consumption were more prevalent among hypertensive subjects, except for smoking. The high prevalence of cardiometabolic risk factors reported in our study is similar to reports by Akintunde et al. among university staff in Nigeria.^20^ Besides factors such as a high-salt diet, low physical activity and high socio-economic status (not examined in our study), these are established risk factors for hypertension, which in itself is a major risk for CVD. Urbanisation, among other determinants, has largely been queried.^8^,^19^,^29^.

Our study showed that all risk factors studied were most prevalent among participants with diabetes. About three out of four diabetic subjects had hypertension. Other studies have reported a high prevalence of high blood pressure among diabetic subjects in Cameroon^30^ and Tanzani,^31^ although lower than in ours.

The higher prevalence of overweight, obesity (abdominal and general) as reflected in WC and mean BMI, hypercholesterolaemia, alcohol abuse and smoking, being more common in diabetic than non-diabetic subjects, is however an expected finding, as they all have individual and associative effects in predisposition to the development of diabetes.^8^,^30^ Therefore, while diabetes in itself has been demonstrated to be an independent cardiovascular risk factor,^32^ the impact of its association or cumulative effect with other traditional risk factors in the development, progression, morbidity and mortality linked with CVDs cannot be overemphasised.

## Limitations and strengths of the study

Our study has several limitations that deserve mention. First the hospital base of the recruitments and the selected nature of the participants could have increased the chances that those included were at high risk for metabolic risk factors, which therefore could account for the high prevalence of cardiometabolic risk factors in our study. Secondly, the method of diagnosis of hypertension could be subject to debate, but it has been clearly evidenced by Burgess et al. that failure to carry out multiple measurements to confirm the diagnosis may lead to false positives.^33^ Thirdly, quantity or concentration of alcohol in the local beer may vary from one country to another, and we could not assess non-industrial alcoholic beverages. Lastly, although the overall sample size was large, the number of patients contributed from each participating centre within the countries tended to be small, therefore precluding meaningful centre-level analysis.

In spite of these limitations, the multi-centre, multi-national character of this study increased our chances of adequately exploring the prevalence of cardiometabolic risk factors in the participating countries, and demonstrating evidence of the growing cardiovascular risk factors in this region plagued with communicable diseases. The use of well-trained data collectors (medical practitioners) also gave confidence in the measured parameters.

## Conclusions

This study reports alarmingly high prevalences of cardiometabolic risk factors among adults presenting at urban and semi-urban hospitals in selected countries in SSA, which is in line with IDF projections of NCDs (hypertension and diabetes mellitus) in the region. It also raises the question of the influence of rapid urbanisation on the development of risk factors for imminent cardiovascular and metabolic diseases. This has considerable public health impact for an already economically disadvantaged setting to design new methods or further strengthen existing measures and interventions for the control of chronic diseases in the region.
